# Deep Brain Stimulation of the Subthalamic Nucleus Induces Impulsive Responses to Bursts of Sensory Evidence

**DOI:** 10.3389/fnins.2019.00270

**Published:** 2019-03-29

**Authors:** Dennis London, Michael H. Pourfar, Alon Y. Mogilner

**Affiliations:** Department of Neurosurgery, Center for Neuromodulation, NYU Langone Health, New York, NY, United States

**Keywords:** DBS, STN, Parkinson's disease, decision-making, drift diffusion model

## Abstract

Decisions are made through the integration of external and internal inputs until a threshold is reached, triggering a response. The subthalamic nucleus (STN) has been implicated in adjusting the decision bound to prevent impulsivity during difficult decisions. We combine model-based and model-free approaches to test the theory that the STN raises the decision bound, a process impaired by deep brain stimulation (DBS). Eight male and female human subjects receiving treatment for Parkinson's disease with bilateral DBS of the STN performed an auditory two-alternative forced choice task. By ending trials unpredictably, we collected reaction time (RT) trials in which subjects reached their decision bound and non-RT trials in which subjects were forced to make a decision with less evidence. A decreased decision bound would cause worse performance on RT trials, and we found this to be the case on left-sided RT trials. Drift diffusion modeling showed a negative drift rate. This implies that in the absence of new evidence, the amount of evidence accumulated tends to drift toward zero. If evidence is accumulated at a constant rate this results in the evidence accumulated reaching an asymptote, the distance of which from the bound was decreased by DBS (*p* = 0.0079, random shuffle test), preventing subjects from controlling impulsivity. Subjects were more impulsive to bursts of stimuli associated with conflict (*p* < 0.001, cluster mass test). In addition, DBS lowered the decision bound specifically after error trials, decreasing the probability of switching to a non-RT trial after an error compared to correct response (28% vs. 38%, *p* = 0.005, Fisher exact test). The STN appears to function in decision-making by modulating the decision bound and drift rate to allow the suppression of impulsive responses.

## Introduction

The STN has been implicating in slowing of decisions, especially those made in the presence of conflicting evidence (Zavala et al., 2015b). Unsurprisingly, DBS of the STN, like dopaminergic medication (Weintraub et al., 2006), may cause impulse control disorders (Hälbig et al., 2009; Castrioto et al., 2014), despite its benefit in improving motor control (Benabid et al., 2009).

Decisions are made by accumulating evidence for potential actions until an evidence bound is reached (Ratcliff and McKoon, 2008). Conflicting evidence results in difficult decisions and slower responses (Frank et al., 2007). While this can occur simply through less net evidence, response slowing may also occur through raising of the decision threshold during times of high conflict. This may be mediated through the STN, which, when lesioned (Baunez et al., 1995; Eagle et al., 2008; Obeso et al., 2014) or stimulated (Frank et al., 2007), is unable to prevent impulsive responses. The STN receives direct input from frontal cortex through the hyperdirect pathway (Monakow et al., 1978; Nambu et al., 2002), and phase coupling of STN and mediofrontal theta activity correlates with the decision bound (Herz et al., 2016). Furthermore, conflict increases theta power (Cavanagh et al., 2011; Zavala et al., 2013, 2014) and neuronal firing (Zavala et al., 2015a) in the STN and theta coherence between the STN and mediofrontal areas (Zavala et al., 2014). However, this activity is reduced when subjects respond quickly despite high levels of conflict, resulting in the hypothesis that the medial prefrontal cortex (mPFC) acts on the STN through the hyperdirect pathway to cause the STN to raise the decision threshold (Frank et al., 2007; Cavanagh et al., 2011).

DBS causes faster responses despite conflicting evidence (Frank et al., 2007), and drift-diffusion modeling has shown a decreased decision bound during conflict (Frank, 2006). While electrophysiological studies modeling the decision bound have used the random dots task, in which subjects accumulate sensory evidence over time (Zavala et al., 2014; Herz et al., 2016), early studies used simultaneous presentation of two individual stimuli with different probabilities of reward to simulate conflict. Two studies using the evidence accumulation paradigm found that DBS decreases the decision threshold (Green et al., 2013; Pote et al., 2016). The stimuli in these studies are either discrete stimuli presented concurrently (Frank et al., 2007; Cavanagh et al., 2011; Zavala et al., 2013, 2015a) or continuous stimuli presented over a time interval (Green et al., 2013; Zavala et al., 2014; Herz et al., 2016; Pote et al., 2016). Humans make decisions using a combination of these paradigms, by integrating discrete stimuli over a period of time, and DBS impairs performance on a task requiring integration of a train of 3 stimuli separated by a constant time interval (Coulthard et al., 2012). Timing of stimuli presented relative to one another can affect decisions, an effect that cannot be modeled in previous paradigms used to assess the effect of DBS on decision-making.

We sought to determine whether DBS of the STN decreases the decision threshold in a sensory accumulation of evidence task in which subjects accumulate discrete information over time. We used a modified version of the Poisson clicks task (Brunton et al., 2013), in which stochastically presented discrete units of evidence sample the space of multiple decision-making parameters, including the decision bound. In addition to modeling the bound, we sought to test behavioral predictions of DBS-induced lowering of the decision threshold on high-conflict trials. To do this we used a task which combined reaction time trial design with non-reaction time trials to assess subjects' performance when they are forced to respond before reaching a decision threshold.

## Methods

### Subjects

Subjects were men and women being treated for Parkinson's disease with bilateral DBS of the STN. They were recruited at their regular appointments and signed informed consent for participation in the study as approved by the NYU Langone Medical Center IRB. All subjects had no clinically significant cognitive or hearing impairments. 7/8 subjects were right-handed, and 4/8 were predominantly symptomatic on the right.

### Experimental Design

Subjects completed 30-min sessions on different days, and took their regularly scheduled medication throughout the study. Each session was completed in two 15-min blocks, DBS OFF, and DBS ON with randomly assigned order. Trials began with a “Ready” phase, which terminated after the subject initiated the trial, followed by a “Stimulus” phase composed of simultaneous click trains played from the left and right headphone, respectively. Subjects were instructed to respond by selecting the side with more clicks, and they were informed that they could respond after the stimulus ended or interrupt the stimulus with a response. The stimulus phase ended either with a subject response (response time (RT) trial) or with offset of the stimulus after a pre-determined time (non-RT trial). Stimulus offset began the “Response” phase in non-RT trials. Subjects were not explicitly informed of stimulus termination or cued for a response in either RT or non-RT trials. Distinct sounds indicated correct or incorrect responses.

The duration of each trial's “Stimulus” phase, *t*_*stim*_ was selected so that subjects could not predict when stimulus offset would occur. It was chosen from the following probability distribution:

(1) $$\begin{array}{r} p\left(t_{stim}\right)=\alpha +\beta e^{-\beta t_{stim}} \end{array}$$

After a fixed time, α, the stimulus had a constant probability of ending at any time defined by the time constant β. Click trains were generated from Poisson distributions with average rates *r*_*low*_ and *r*_*high*_. Such that:

(2) $$\begin{array}{r} r_{low}+r_{high}=20\;Hz. \end{array}$$

(3) $$\begin{array}{r} \gamma =log_{10}\left(\frac{r_{high}}{r_{low}}\right) \end{array}$$

The parameters α, β, γ, were adjusted for each subject between sessions with the goal of subjects achieving 70% accuracy with half of trials being RT trials. Subjects were compensated for each session as well as each correct trial, encouraging subjects to complete more trials while maintaining accuracy.

At the end of each 15-min block stimulators were turned off/on. There was no washout period between blocks. Subjects were allowed to complete the task either in the office or at home.

### Statistical Analysis

All data were analyzed from 8 out of 13 subjects who completed at least 2 sessions, so that a minimum amount of data was available for modeling. Non-RT trials with response time above 5 s were discarded as timeouts (114/5,199 total trials). All data was analyzed in Matlab R2017a.

#### Model Fitting

We fit a drift-diffusion model to subject responses based on each discrete click as a unit of evidence using the method of Brunton et al. (2013) as described in brief here. Parameters for the full 9-parameter model are summarized in Table 1. Click value, *C(t)*, was altered by sensory noise by multiplication by a factor of η, drawn from a normal distribution with mean 1 and variance σ_*s*_^2^. The accumulated evidence was subject to an exponential drift toward or away from 0 described by the parameter λ. Diffusion was defined by a Wiener process with variance σ_*a*_^2^. Overall, evidence accumulated, *a*, was described by the following equation:

(4) $$\begin{array}{r} da=\{\begin{array}{cc} 0 & if\;|a|\geq B\\ \sigma_{a}dW+C\left(t\right)\left(\eta_{R}{\delta_{t,t}}_{R}-\eta_{L}{\delta_{t,t}}_{L}\right)dt+\lambda adt & otherwise \end{array} \end{array}$$

where *dW* is a Weiner process and *B*, is the decision bound. _δ_*t, t*_*R*/*L*_ are delta functions equal to 1 at the time of right or left clicks. The initial condition of *a* was defined by a normal distribution with variance $\sigma_{i}^{2}$. *C(t)* was subject to adaptation with an adaptation parameter, ϕ, and time constant, τ_ϕ_:

(5) $$\begin{array}{r} \frac{dC}{dt}=\frac{1-C\left(t\right)}{\tau_{\phi }}+\left(\phi -1\right)C\left(t\right)\left(\delta_{{t,t}_{R}}+{\delta_{t,t}}_{L}\right) \end{array}$$

**Table 1:** Parameters fit in drift-diffusion models in this study. The final model selected using AIC analysis used only 7 parameters (see Figure 3).

**Model Parameters**
λ: exponential drift to or from 0
*σ_*i*_*: accumulator initial value noise
*σ_*a*_*: accumulator noise
*σ_*s*_*: sensory noise
*B*: bound
ϕ: sensory adaptation
*τ_ϕ_*: sensory adaptation time constant
*bias*: constant shift of initial accumulator value
*L*: rate at which subjects respond randomly

An additional bias parameter was used, as well as a lapse parameter *L*, which represented the probability of a subject producing a random response.

We reduced the number of parameters from 9 to 7 using AIC analysis (**Figure 3B**), such that in our final model, evidence accumulated was governed by the following equation:

(6) $$\begin{array}{r} da=\{\begin{array}{cc} 0 & if\;|a|\geq B\\ \sigma_{a}dW+C\left(t\right)dt+\lambda adt & otherwise \end{array} \end{array}$$

In Brunton et al., all trials were non-RT and bounds could not be adequately fit for many subjects. Therefore, we fit the decision bound by maximizing the log-likelihood of each subject's fraction of RT trials. The probability of not responding exactly at time *t*_*i*_ is $p_{i}^{nRT}$, the probability mass not at the bounds. The probability of an RT trial was calculated as the probability of responding 750 ms (non-decision time, 99.9% of non-RT trials occurred after this time) before stimulus end.

(7) $$\begin{array}{r} p^{RT}=\sum \left[\left(1-\overset{nRT}{\underset{i}{p}}\right)\overset{i-1}{\underset{1}{\prod }}p_{j}^{nRT}\right] \end{array}$$

The fitted bound confidence interval was calculated using the 95% confidence interval of the fraction of RT trials. *p*^*RT*^ values were tested for *B*∈[0, 50].

Model identifiability was confirmed by simulating responses using 10 separate parameter sets and using these simulated responses to generate new best-fit parameters. The parameter sets were used to simulate responses to trials performed by subject 1 in the DBS OFF condition. As in Brunton et al. (2013), to make comparisons across different parameters, the parameters were all normalized such that each of the original parameters ranged from 0 to 1.

#### Impulsivity Index

To calculate the impulsivity index, we selected RT trials with bursts of *n* same-sided clicks at particular frequencies. The click train corresponding to the response on each trial was analyzed for bursts. Burst frequencies were defined based on the times of the first and last clicks, *t*_1_, and *t*_*N*_, respectively: $f=\frac{n}{t_{N}-t_{1}}=\frac{n}{\Delta t}$. We discretized Δ*t* into overlapping bins defined as

(8) $$\begin{array}{r} n\left(i-1\right)*\;5\;ms<\Delta t_{i}<n\left(i-1\right)*5\;ms+n\;*\;25\;ms \end{array}$$

This created bins of identical frequency bands for varying *n* and *i*. For all RT trials containing at least 1 burst of *n* clicks in Δ*t*_*i*_, the burst response time, *t*_*B*_, is the time from the beginning of the last burst until the subject's response.

If subjects accumulate clicks without regard for when they are presented relative to one another, then *t*_*B*_ is a randomly distributed variable that depends only on the generating Poisson process. We can reformulate each click train as a sequence of its inter-click intervals (ICI). Rearranging the ICIs creates a new trial that should have an identical response time if click timing is irrelevant to subjects. In contrast, impulsively responding subjects would respond differently to the rearranged trial. Considering the non-impulsive subject, we generated the expected distribution of burst reaction times. For each trial, we shuffled the ICIs and selected the last burst of *n* clicks in Δ*t*_*i*_ to calculate the shuffled trial's burst response time, $t_{B}^{s}$. This was done 1000 times for each trial to calculate the expected distribution of *t*_*B*_. The impulsivity index is defined for *[n*, Δ*t*_*i*_*]* as the fraction of observed *t*_*B*_ faster than 95% of the expected *t*_*B*_. A non-impulsive subject would have an impulsivity index of 0.05.

Statistical significance was assessed by adapting the non-parametric cluster mass method (Maris and Oostenveld, 2007) to the chi-square tests for goodness of fit and independence. The cluster mass method allows statistical testing of time series data with correction for multiple comparisons by taking advantage of the correlated nature of time-adjacent points. We applied the same methodology to “frequency-series” data, since frequency-adjacent points are similarly correlated. The non-parametric cluster mass test uses shuffling of the data between compared series to generate an expected distribution. This is unnecessary here since the impulsivity index is a proportion and follows a binomial distribution. Statistical testing can therefore be done using a χ^2^ distribution.

To determine whether the OFF and ON impulsivities were different from 0.05, χ^2^ from the expected value of 0.05 was used. To compare the OFF and ON impulsivities, χ^2^ of a contingency table composed of the OFF and ON proportions was used. Frequency-adjacent χ^2^ corresponding to *p* < 0.05 (one degree of freedom) were used to form clusters, and each cluster's test statistic is the sum of its individual χ^2^ values. Significant clusters were defined as those with test statistics (i.e., cumulative χ^2^) greater than that corresponding to a critical *p*-value for the χ^2^ distribution with degrees of freedom equal to the size of the largest cluster. Given that we performed several of these cluster mass tests we were conservative by selecting our critical *p*-value as 0.001. Each cluster mass test allows for comparison of impulsivity at many different frequencies while controlling the false alarm rate. The use of *p* < 0.05 to select clusters controls the sensitivity of the test, not the false alarm rate.

## Results

**Figure 1:**
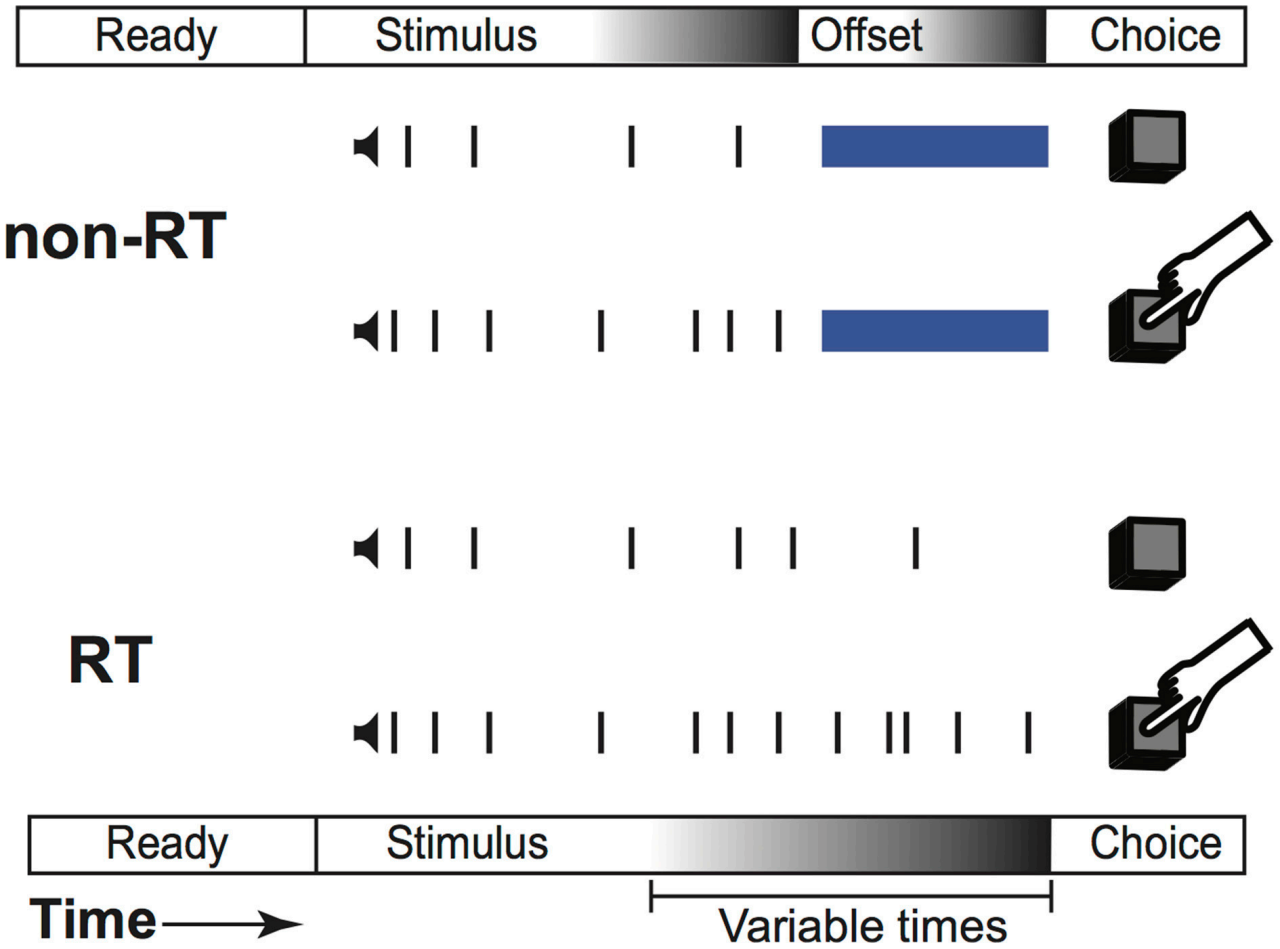
Task structure. Structure of the same trial displayed as an RT trial or a non-RT trial.

Our task is a two-alternative forced choice task that fused reaction time (RT) and non-RT trial design. As diagrammed in Figure 1, subjects listened to two simultaneous trains of “clicks,” one from each headphone. These clicks were generated from underlying Poisson distributions with different rates. Subjects were instructed to respond with the side with more clicks when they were confident of their choice. A Poisson process governed trial termination; therefore, at any point in time, the current stimulus duration provided no information regarding stimulus termination time, preventing the creation of an urgency signal. Subjects were not informed of stimulus termination; they were simply instructed to respond when ready. This naturally created two sets of trials, those in which the subjects responded before (RT trials) and after (non-RT) stimulus offset. On RT trials, subjects responded when they hit their decision threshold, while on non-RT trials, they were forced to respond before reaching their threshold.

**Figure 2:**
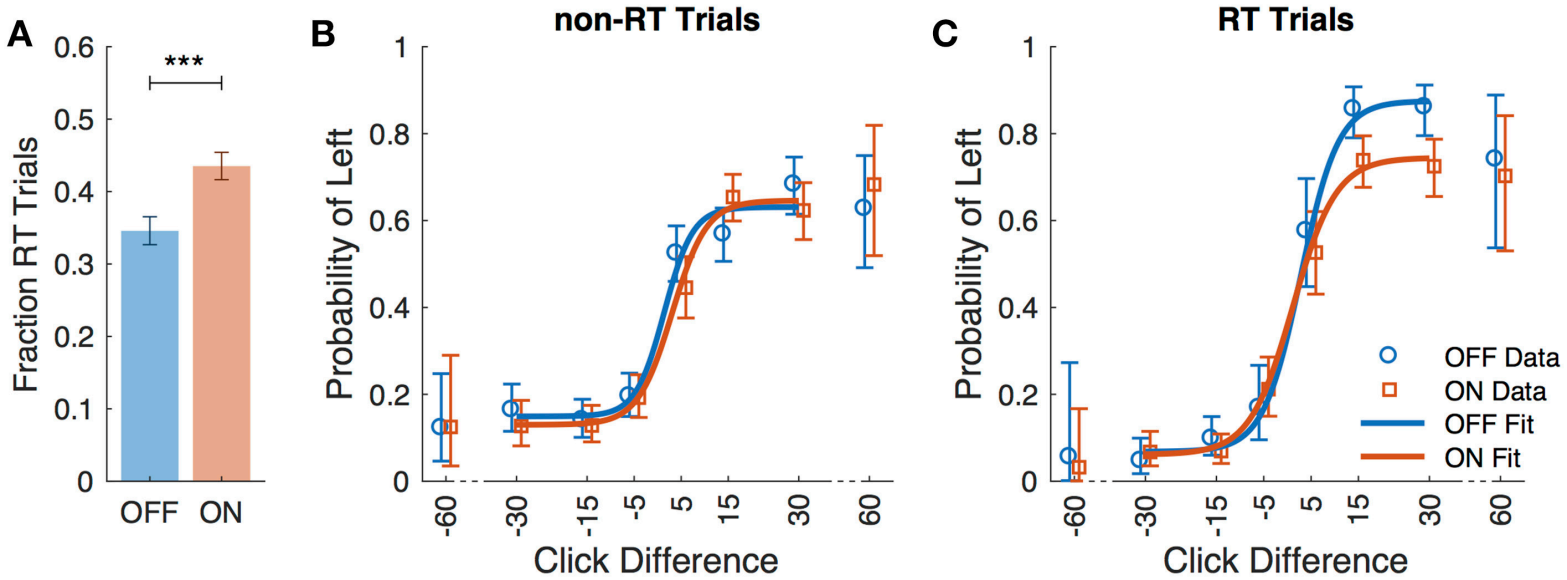
Performance data across all subjects. **(A)** Fraction of trials in which subjects responded prior to termination of stimulus (RT trials) for DBS OFF (blue) and ON (red). Subject responses as a function of click difference (positive is more left clicks) for non-RT trials **(B)**, and RT trials **(C)**. Markers represent data, and curves are the logistic fit. Error bars are 95% confidence intervals. ^***^P < 0.001.

Eight subjects completed at least 2 sessions for a total of 5,085 trials. All subjects took dopaminergic medication at their regularly scheduled doses while participating in this study. Their medications at the time of task completion as well as the stimulation settings used are shown in Table 2. See Table 3 for full subject data regarding task completion. Subjects were more accurate on RT trials (RT accuracy: 80.0%, non-RT accuracy: 69.1%, *p* < 10^−5^, Fisher exact test) In the DBS OFF condition subjects responded before stimulus offset (RT trials) on 34.6% of trials, and when DBS was turned ON this rate increased to 43.5% (*p* < 10^−5^, Fisher exact test). This increased rate of early response decreased accuracy on the trials where the correct answer was a leftward response (OFF: 80.4%, ON: 69.7%; *p* = 1.82 ^*^ 10^−4^, Fisher exact test), as shown in Figures 2A–C. A multinomial logistic regression using RT trials with random effects subject terms and fixed effects for correct side, symptomatic side, and for DBS status showed a significant accuracy effect only for the correct side × DBS interaction term (odds ratio: 0.42, 95% confidence interval: 0.26–0.69). Odds ratios and significance for all model terms are shown in Table 4.

**Table 2:** Medications and DBS protocol during completed sessions for each subject.

**Patient summary**				
**Subject**	**Sessions**	**Medications**	**Left lead**	**Right lead**
1	1, 2	carbidopa-levodopa, ropinirole	1+2– 3.0/90/60	0+1– 1.7/90/130
2	1	carbidopa-levodopa-entacapone	1+0– 2.7/90/145	C+1– 2.4/60/130
	2	carbidopa-levodopa-entacapone	1+0– 3.1/60/130	C+1– 2.4/60/130
3	1, 2, 3, 4	carbidopa-levodopa	1+2– 3.2/90/145	1+2– 3.5/90/185
4	1	carbidopa-levodopa	C+1– 2.0/60/130	C+1– 2.4/60/130
	2	carbidopa-levodopa	C+0–1– 2.8/60/145	C+1– 2.6/60/145
5	1	carbidopa-levodopa	3+0–1– 2.2/90/120	3+2–1+ 2.5/60/130
	2	carbidopa-levodopa-entacapone, rasagiline	3+0–1– 2.2/90/120	3+2–1+ 2.8/60/130
6	1	carbidopa-levodopa, rotigotine, amantadine	C+2–3– 3.5/90/60	C+3– 3.0/60/60
	2, 3	carbidopa-levodopa, rotigotine	C+2–3– 2.8/90/130	C+3– 2.8/60/130
7	1	carbidopa-levodopa, ropinirole	C+1– 3.0/60/185	2+1– 3.5/210/60
	2	carbidopa-levodopa	C+1– 3.0/60/185	2+1– 3.5/210/60
8	1, 2	carbidopa-levodopa-entacapone, ropinirole, rasagiline	1+2– 4.2/60/145	1+2– 4.0/60/145

*Stimulation settings indicate the contacts used as the cathode (“+”) and anode (“–”) followed by “amplitude (mV)/pulse width (μs)/frequency (Hz)”. “C+” indicates monopolar instead of bipolar stimulation. Contacts are numbered from 0 to 3*.

**Table 3:** Data summary.

**Data collection summary**							
**Subject**	**Dominant side**	**Symptomatic side**	**Trials**	**Sessions**	**Alpha**	**Beta**	**Gamma**
1	Right	Right	635	2	1, 2	2.5, 3	0.4
2	Left	Right	664	2	2, 1	3, 2.5	0.4
3	Right	Left	1,052	4	0.5	1.5	0.4
4	Right	Left	600	2	0.5, 1	1.5, 2	0.4
5	Right	Right	512	2	0.5, 1	1.5, 2.5	0.4, 0.3
6	Right	Right	537	3	1.5, 1.5, 0.5	3, 3, 1.5	0.4
7	Right	Left	673	2	1.5, 0.5	3, 1.5	0.4
8	Right	Left	412	2	1	2.5	0.4

*The dominant side, symptomatic side, number of trials, sessions, and corresponding parameters for each session are shown for each patient*.

**Table 4:** RT trials logistic regression.

**RT Trials Logistic Regression**			
	**Odds ratio**	***t***_**(1993)**_	***P*****-value**
Side	0.99 (0.63, 1.55)	−0.051	0.96
Symptoms	1.01 (0.25, 4.15)	0.017	0.99
DBS	1.27 (0.82, 1.98)	1.08	0.28
Side × symptoms	0.67 (0.42, 1.07)	−1.67	0.096
Side × DBS	0.42 (0.26, 0.69)	−3.48	5.0E-04
Symptoms x DBS	0.90 (0.55, 1.47)	−0.42	0.67

*Odds ratios with 95% confidence intervals (in parentheses) and p-values for model terms for the multinomial logistic regression on accuracy for RT trials. Odds ratios are for left to right for each term*.

**Figure 3:**
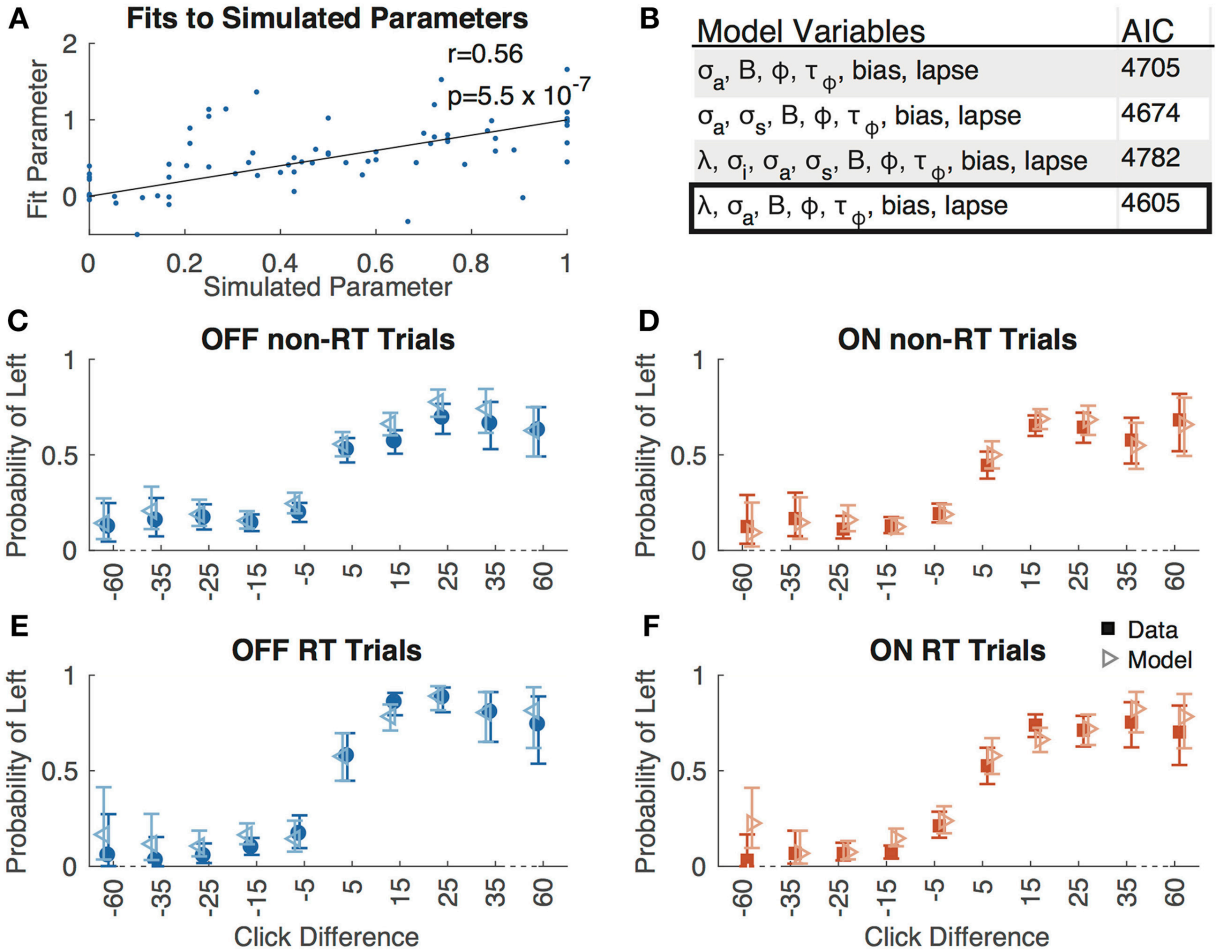
Model validation. **(A)** Best-fit parameters to responses simulated using 10 parameter sets plotted against the simulated parameters. All parameters are normalized such that the simulated parameters are between 0 and 1. The line of equality is shown along with the Pearson's correlation coefficient (r) and associated *p*-value. **(B)** AIC values for 4 different drift-diffusion models with the parameters shown. Box indicates the model we selected. Accuracy as a function of click difference for non-RT OFF **(C)**, ON **(D)**, RT OFF **(E)**, and ON **(F)** trials for actual data (solid boxes) and responses simulated using best-fit parameters (empty triangles).

**Figure 4:**
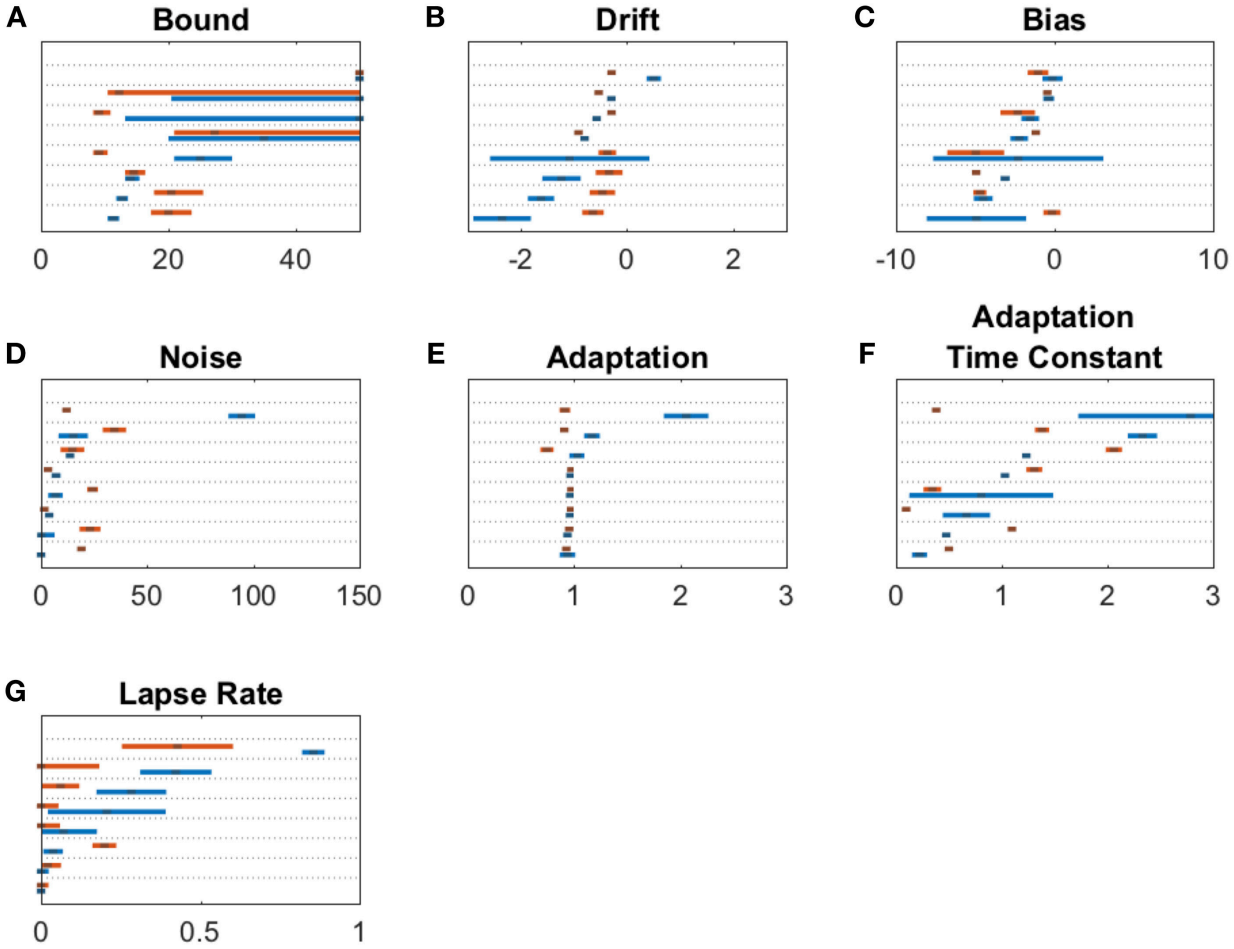
Drift diffusion parameter estimates for bound **(A)**, drift **(B)**, bias **(C)**, noise **(D)**, adaptation **(E)**, adaptation time constant **(F)**, and lapse rate **(G)**. Maximum likelihood parameters for each subject for DBS OFF (blue) and ON (red). Parameters on each panel are sorted from least to greatest DBS OFF parameter with each subject's DBS ON parameter adjacent to its corresponding OFF parameter. Error bars are 95% confidence intervals. Non-overlapping 95% confidence intervals within the same subject for DBS ON vs. OFF indicate a significant parameter change. In **(A)**, error bars stretching to the end of graph indicate confidence interval fits that did not converge.

**Figure 5:**
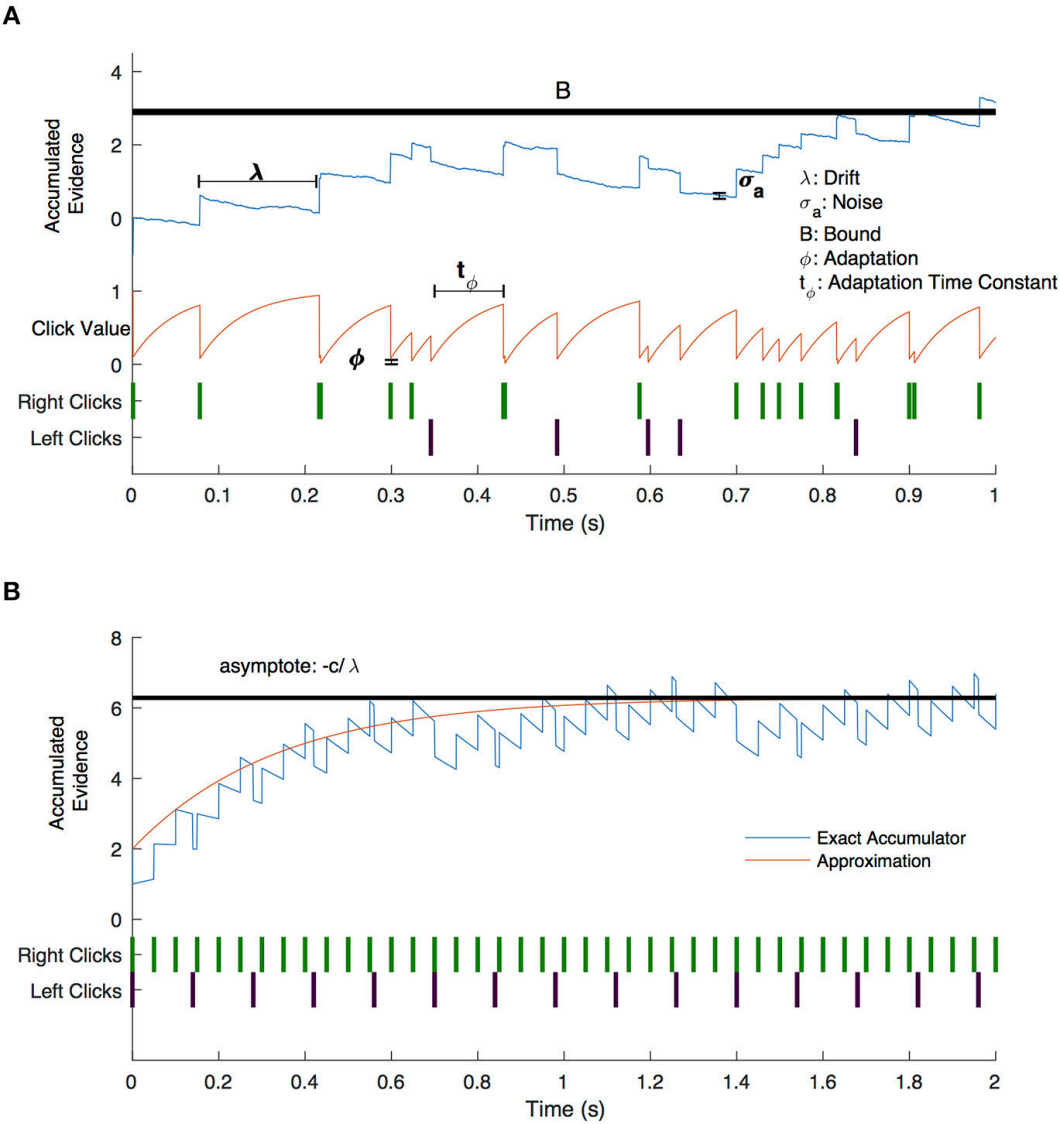
Drift-diffusion model example trial. **(A)** A visual description of the drift diffusion model of accumulated evidence (blue line). Right (green) and left (purple) click times are shown as a raster plot. The clicks that occur soon after prior clicks have a value (red line) that is adjusted by the adaptation (ϕ) and adaptation time constant (t_ϕ_) parameters. Accumulated evidence drifts with parameter λ and is affected by noise (σ_a_). Not shown are the bias parameter which shifts the positive or negative bound without shifting both and the lapse parameter which governs the frequency with which subjects respond randomly. **(B)** Drift diffusion model with constant click rates (blue line) and approximate click value assuming continuous evidence presentation (red line). Evidence approaches an asymptote for negative values of λ.

We fit a 7-parameter drift-diffusion model, modified from Brunton et al. (2013), to the data from each subject, separately for DBS OFF and ON trials (but with a single parameter set for RT and non-RT trials). Simulated responses generated from model parameters were indistinguishable from the actual data for both non-RT and RT trials, as shown in Figures 3C–F. To validate the model, we used 10 different parameter sets to generate simulated responses to the trials of a single subject in the DBS OFF state (to determine if a single subject's trials were sufficient for fitting), to which we fit the model, recovering parameter fits that reasonably approximated the generating parameters (Pearson's *r* = 0.56, *p* = 0.55 ^*^ 10^−7^), as shown in Figure 3A. We fit 3 additional models using up to the 9 parameters used by Brunton et al., and selected our model based on AIC (Figure 3B). For one subject, the upper and lower bounds of the decision threshold could not be fit. Figure 4 shows the best-fit parameters, and Figure 5A illustrates the effect of the parameters on the accumulation of evidence. Despite prior evidence that DBS decreases the decision bound, only 2/8 subjects had significantly lower decision bounds with DBS ON (Figure 4A). 2/8 subjects had a significantly higher decision bound with DBS ON, and the remainder showed no significant difference. Consistent with the behavioral results (Figures 2B,C) 5/8 and 7/8 subjects had a significant rightward bias (negative bias parameter) with DBS OFF and ON, respectively. There were no differences between DBS ON and DBS OFF in mean decision bound or mean bias across patients (*p* = 0.23, 0.95, respectively, paired random shuffle test). There was a trend in which DBS caused higher decision bounds and less negative drift parameters in some patients. Negative drift parameters, present in 7/8 and 8/8 patients in DBS OFF and ON, respectively, result in asymptotes of the total accumulated evidence (patient 8 was not used in this analysis due to a positive drift parameter). Considering the simplified case of no diffusion, a constant rate of evidence accumulation (the difference between average left and right click rates), *c*, and a drift parameter, λ, the rate of change of the total accumulated evidence is

(9) $$\begin{array}{r} \frac{da}{dt}=c+\lambda a \end{array}$$

**Figure 6:**
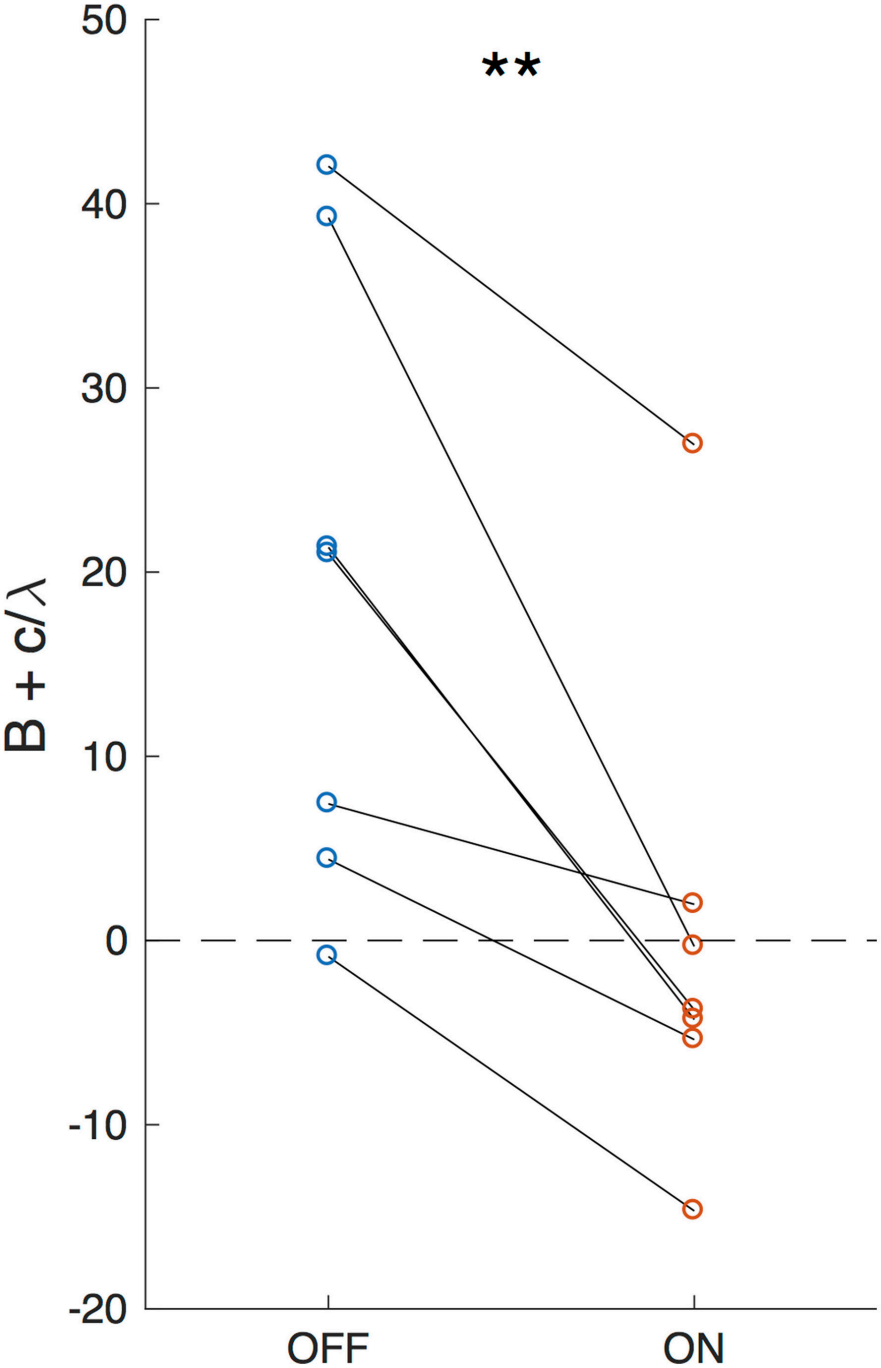
Effective decision bound. The difference between the evidence asymptote and the bound (B+c/λ) for DBS OFF and ON. Lines connect data points from the same patient. ^**^P < 0.01.

Setting $\frac{da}{dt}=0$, results in the accumulated evidence asymptote: $-\frac{c}{\lambda }$, as illustrated in Figure 5B. The closer this value is to the decision bound, the more likely is a decision caused by stochastic variables (accumulator noise, variation in click rates, etc.). Thus, the parameter that controls susceptibility of decision-making to randomness is a modified decision bound: $B-\left(-\frac{c}{\lambda }\right)=B+\frac{c}{\lambda }$. As shown in Figure 6, DBS significantly decreased this parameter across patients (*p* = 0.0079, random shuffle test). A negative value (1 patient with DBS OFF and 5 patients with DBS ON) implies an asymptotic evidence value above the decision bound, meaning that there is effectively no asymptote. This results in subjects affected by bursts of evidence, since the asymptote effectively causes subjects to “forget” bursts of evidence and prevents impulsivity.

**Figure 7:**
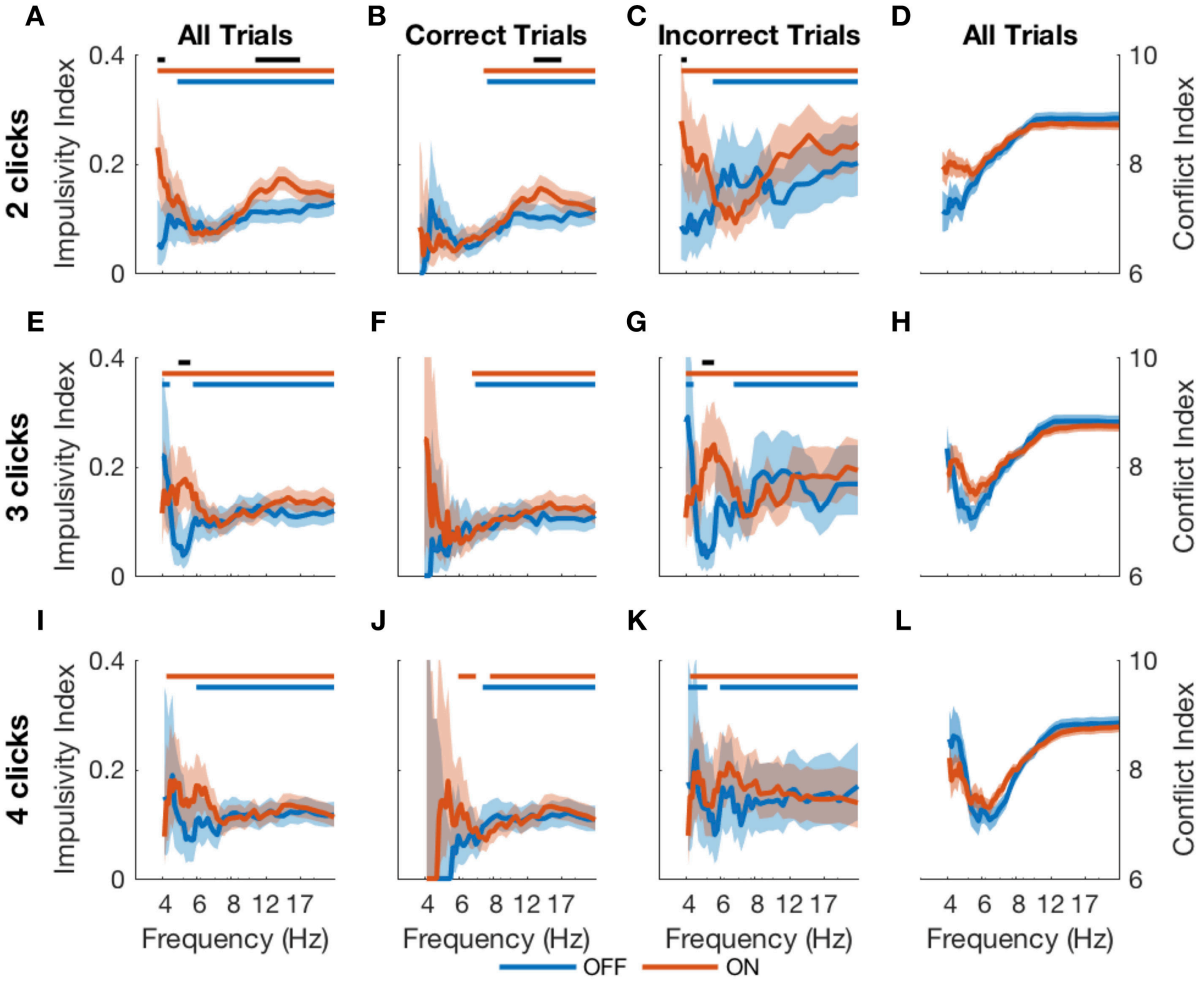
Impulsive responses to bursts. Impulsivity index (higher is more impulsive) calculated for 2- **(A–C)**, 3- **(E–G)**, and 4-click **(I–K)** bursts of particular frequencies for all RT trials (first column), correct RT trials (second column), and incorrect RT trials (third column). The conflict index (difference in click frequency between left and right, higher is lower conflict) for different frequencies is plotted for 2-, 3- and 4-click bursts in the last column **(D,H,L)**. Shading is 95% confidence intervals for impulsivity index and standard error for conflict index. Blue and red bars at the top of each plot indicate statistical significance (from 0.05, which is the expected value if there is no impulsivity) of DBS OFF and ON impulsivity indices, respectively. Black bars indicate significant differences between DBS OFF and ON indices. Statistical significance is *p* < 0.001 using the cluster mass method for the χ^2^ test.

An evidence asymptote implies a different treatment of bursts of clicks than of clicks presented with arbitrary inter-click intervals. We next sought to determine in a model-free way if DBS caused subjects to respond differently to bursts of evidence. Using bootstrapping (see Methods), we calculated an impulsivity index at each frequency in response to 2-, 3-, and 4-click bursts for DBS OFF and ON (Figure 7). An impulsivity index of 0.05 implies a non-impulsive subject. With DBS OFF, subjects were impulsive at all frequencies except those in the 4–8 Hz range. In contrast, with DBS ON, subjects were impulsive in all frequency ranges including the 4–8 Hz range, and for 2- and 3-click bursts, impulsivity was significantly greater than with DBS OFF. This increased impulsivity was explained entirely by incorrect trials: with DBS ON, subjects' impulsivity was both significant and significantly greater than with DBS OFF below 8 Hz on 2- and 3-click bursts.

**Figure 8:**
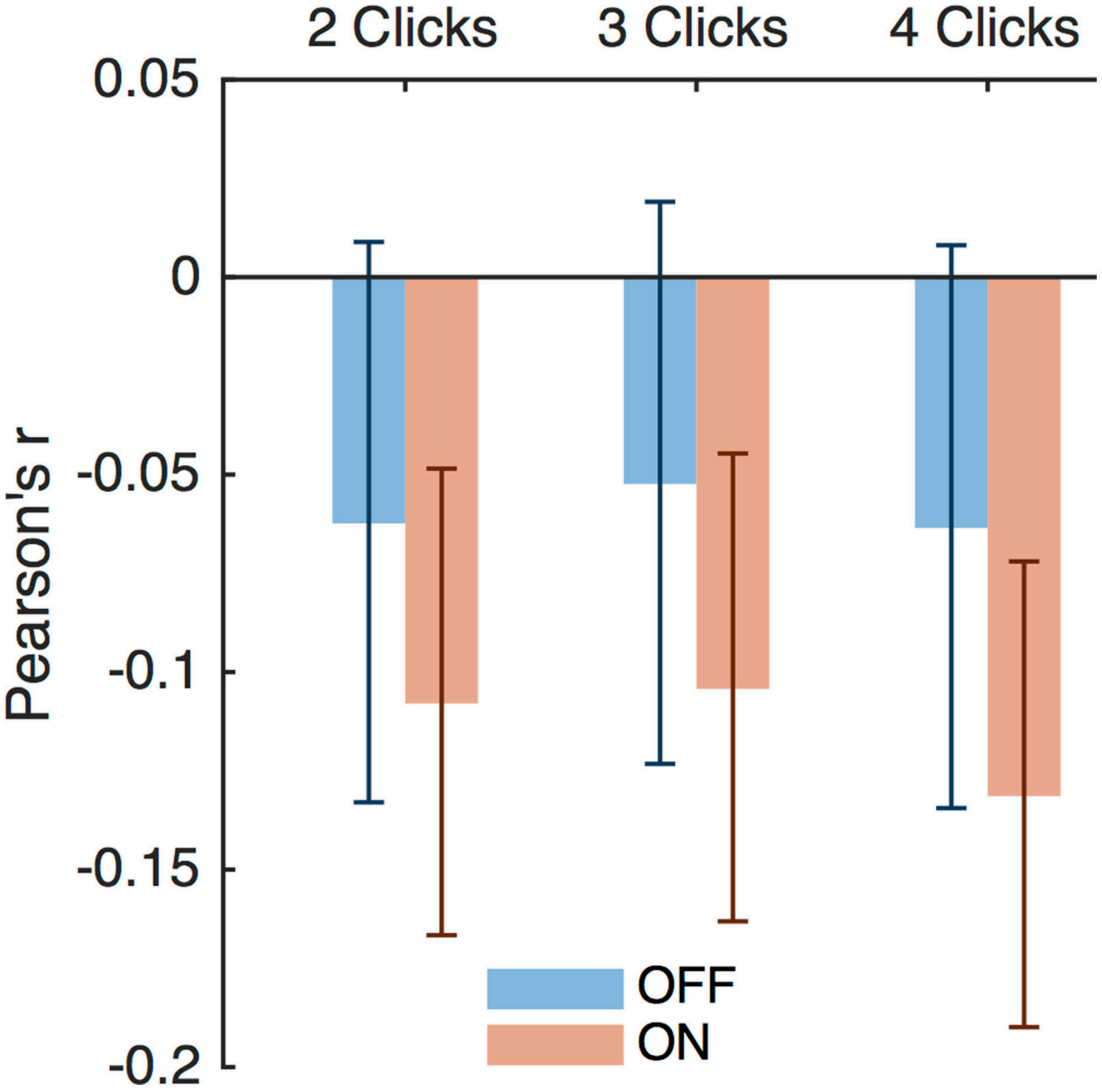
Impulsivity and conflict correlations. Pearson's *r* for impulsivity index and conflict index for trials with 2-, 3-, and 4-click bursts of any frequency. Error bars are 95% confidence intervals.

Thus, DBS caused subjects to respond impulsively and possibly incorrectly to bursts of evidence below 8 Hz. Previous studies have suggested that DBS interferes with modulation of impulsivity at times of high conflict (Frank et al., 2007; Cavanagh et al., 2011). In our task, two opposing click trains create conflict, and the difference between the numbers of clicks per second presented to each side is a natural measure of conflict; a small difference implies high conflict. This is separate from the click bursts themselves as this considers all clicks heard by a subject in a trial, as opposed to a 2-, 3-, or 4-click bursts. Considering all RT trials together showed that the correlation between impulsivity and conflict is small as shown in Figure 8 and is not significantly different between DBS OFF and ON (*p* = 0.22, 0.26, and 0.084, 0.053 for trials with 2-, 3-, and 4-click bursts and across all bursts, respectively, *Z*-test). Examining only trials containing bursts of clicks at specific frequencies, as above, we found empirically that trials with bursts in the 4–8 Hz range contain higher levels of conflict than all other trials (Figures 7D,H,L) (*p* < 10^−4^, random shuffle test for mean conflict in the 4–8 Hz range compared to outside of it, tested separately for 2-, 3-, and 4-click bursts for DBS OFF and ON). Therefore, subjects decrease their impulsive responses to specific bursts of evidence, which signify high levels of conflict, but DBS prevents this decrease, causing paradoxically faster responses when there is conflicting information.

**Figure 9:**
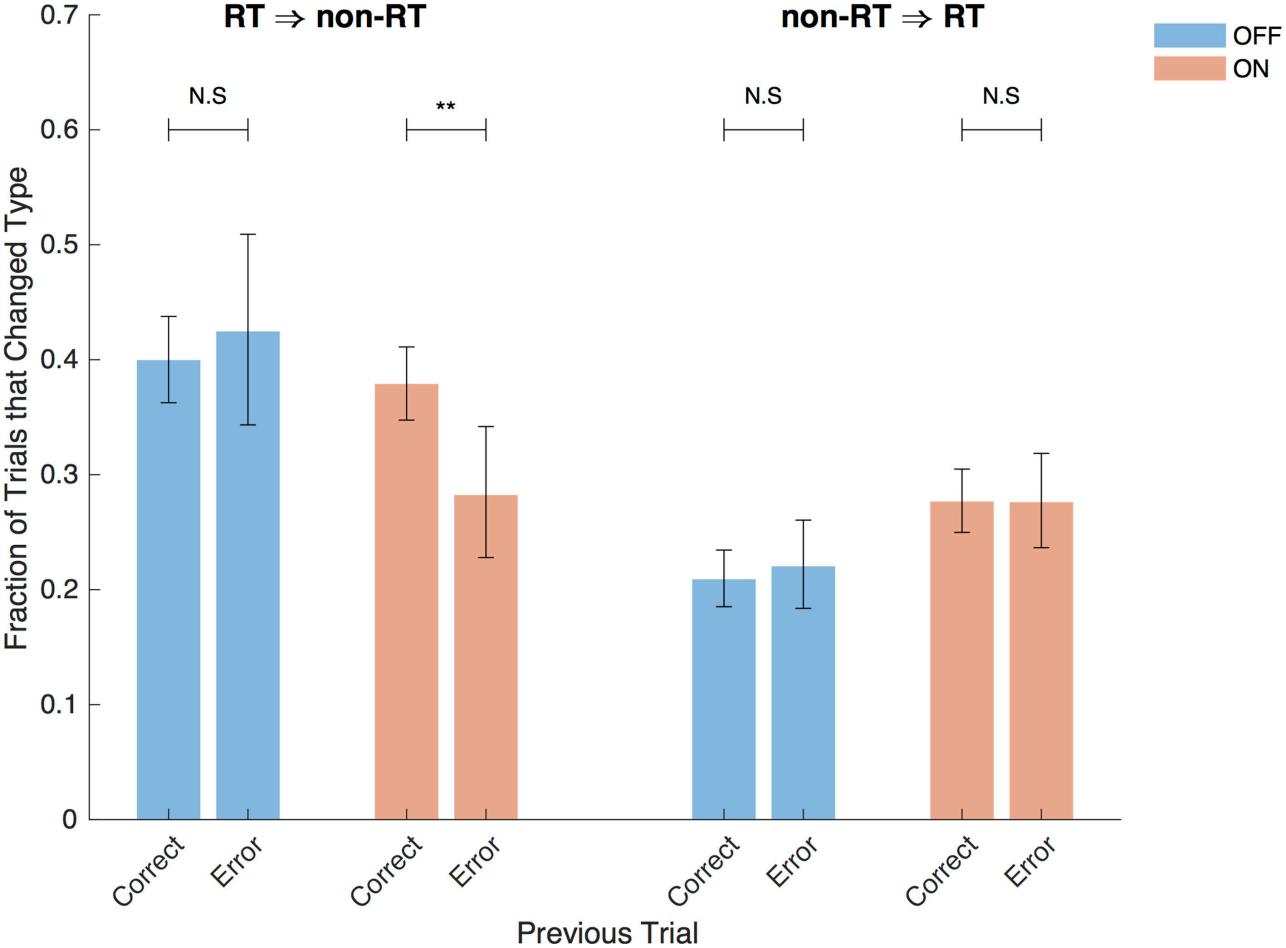
Post-error trials. Fraction of trials after RT trials that were non-RT trials and vice versa split by the accuracy of the previous trial and by DBS status. Error bars are 95% confidence intervals. ^**^P < 0.01.

The STN, in addition to containing electrophysiological correlates of the decision threshold, also contains correlates of post-error slowing (Cavanagh et al., 2014; Siegert et al., 2014; Zavala et al., 2016), suggesting that post-error slowing is accomplished by a dynamic decision threshold that increases after errors. Varying levels of evidence were presented in each trial in this study, so simple slowing of reaction time after an incorrect trial is not to be expected here. However, examining the aftermath of post-error trials in which subjects reached their decision threshold is still informative. With DBS OFF, after correct and incorrect RT trials, the following trial was a non-RT trial in 40% and 42% of cases, respectively (*p* = 0.58, Fisher exact test) as shown in Figure 9. In contrast, the DBS ON condition resulted in a decreased probability of switching to a non-RT trial after an error (38% vs. 28%, *p* = 0.0050, Fisher exact test); this suggests that subjects were actually more likely to adjust their decision threshold downward after an error in which they hit their decision threshold. There was no difference in trial-switching after incorrect non-RT trials (*p* = 0.64, *p* = 1, DBS OFF and ON, respectively, Fisher exact test). While this analysis combines trials from all subjects, similar results are obtained when taking into account different subjects using a mixed effects multinomial logistic regression with random effects terms for the subjects and a fixed effect term for the accuracy of the prior trial. With DBS ON, the accuracy of the prior trial is a significant predictor of the next trial being non-RT after an RT trial (*p* = 0.037), but it is not a significant predictor with DBS OFF (*p* = 0.92).

## Discussion

Using a two-alternative forced choice perceptual decision-making task with both RT and non-RT trials, we found that DBS of the STN does more than simply decrease the decision bound. DBS causes a decrease in accuracy on RT trials but not non-RT trials, as would be expected if DBS lowers the decision bound, but drift-diffusion modeling showed no consistent DBS-mediated changes on decision bound or drift, individually. Instead it showed that the combined effects on these two parameters result in a decreased difference between an evidence asymptote and the decision bound. In 4/7 patients, DBS abolished the evidence asymptote altogether, effectively preventing the filtering of bursts of evidence. Model-free analysis showed that subjects were more impulsive to bursts of evidence with an intrinsic frequency below 8 Hz.

Neural correlates of conflict are present in the STN on both perceptual (Zavala et al., 2013, 2014, 2015a) and probabilistic reward (Frank et al., 2007; Cavanagh et al., 2011) decision-making tasks. The STN also contains the correlate of the decision bound (Herz et al., 2016), and DBS reduces decision threshold in perceptual (Green et al., 2013; Pote et al., 2016) and probabilistic reward (Frank et al., 2007; Cavanagh et al., 2011) tasks. While a DBS-induced decrease of the decision bound is not inconsistent with our data (and was found to occur in 2/8 subjects using drift-diffusion modeling), it is not sufficient to explain all of our results. In contrast to previous tasks used to assess decision-making in the STN, ours uses many discrete stimuli presented in conflict with one another over a time interval. Therefore, the effect of the temporal variation of stimuli can be assessed.

Using this feature of our task, we show that DBS of the STN abolishes the filtering of bursts of evidence. This results in impulsive and incorrect choices to particular temporal patterns of evidence. With DBS OFF, subjects respond impulsively to bursts except those with frequencies <8 Hz. DBS prevents modulation of this impulsivity and causes impulsive responses to bursts of all frequencies resulting in incorrect responses to bursts <8 Hz, which included trials with the greatest level of conflict in this task. This is consistent with prior behavioral evidence that DBS of the STN causes impulsive and incorrect choices (Frank et al., 2007; Cavanagh et al., 2011) as well as the presence within STN of the neural signature of conflict (Zaghloul et al., 2012; Zavala et al., 2015a). Appropriate responses on high conflict trials require coherence of the theta phase between the mPFC and STN, synchronization that appears to be driven by mPFC activity (Zavala et al., 2013, 2014). While this mPFC activity may raise the effective decision bound on high conflict trials, this study suggests that this may occur through a combination of modifications to decision bound and drift rate. We did not find DBS-induced increased impulsivity on trials with increased conflict, but instead found DBS-induced impulsivity to stimuli that were commonly associated with conflict. Thus, DBS of the STN may interfere with the proper use of priors to suppress responses to particular stimuli.

Parkinson's subjects on dopaminergic medication are less able to incorporate priors in their decision-making relative to controls on a perceptual decision-making task (Perugini et al., 2016). The latter study also showed decreased use of priors on the symptomatic side compared to the asymptomatic side. All data presented here is from subjects taking dopaminergic medication, and our results displayed laterality, too; there was a general rightward response bias detectable in both the behavioral response curves and in the modeled bias parameter. However, there was no bias in the behavioral responses to RT trials with DBS OFF (Figure 2C, Table 4). This suggests that the bias is not a feature of our task but is present in subjects' decision-making, and incomplete evidence (e.g. as on non-RT trials) or impulsivity as caused by DBS unmasks this bias. It is possible that bias is introduced by dopaminergic medication, but this was not assessed here since all subjects were on such medication (Table 2). Of note, in Brunton et al., both rodents and non-Parkinsonian human subjects showed little if any bias to one side. DBS caused a decrease in accuracy on RT trials where the correct response was to the left. However, there was no relationship between our subjects' symptomatic side and the reduction in accuracy. It is possible that subjects in this task use certain inter-click intervals to calculate a prior that a trial contains high levels of conflict. They then suppress pre-potent responses not by raising the decision threshold, but by using a negative drift rate, a process that may be suppressed by DBS of the STN. Consistent with this hypothesis, beta band activity in the STN is associated with the inhibition of responses during motor planning (Kühn et al., 2004; Ray et al., 2012; Alegre et al., 2013), and STN DBS prevents motor suppression on go/no-go tasks (Hershey et al., 2010; Wylie et al., 2010; Georgiev et al., 2016).

This is in contrast to the theta phase activity in the STN associated with conflict and raising of the decision bound. Theta coherence (Zavala et al., 2016) and LFP amplitude (Siegert et al., 2014) increase after errors. This activity is associated with post-error slowing (Cavanagh et al., 2014), and we show that DBS of the STN actually speeds post-error responses; subjects were more likely to hit their decision threshold after an error than after a correct response under DBS. Errors due to impulsivity were more likely to occur on trials with bursts in the <8 Hz range, which overlaps with the theta band. However, this band also overlaps with the frequencies of bursts on trials with the highest conflict. Disentangling whether DBS causes impulsivity specifically on bursts in the theta range or on bursts associated with conflict requires varying the frequencies of bursts on the highest conflict trials.

Furthermore, there are elements of decision-making in Parkinson's that we do not address here, such as the effect of dopaminergic therapy on decision-making parameters. Active contact location within STN as well as trajectory through surrounding brain tissue also influences the motor (Koivu et al., 2018) and non-motor (Tsai et al., 2007; York et al., 2009; Witt et al., 2013) effects of DBS in both observational and modeling (Mandali et al., 2015; Mandali and Chakravarthy, 2016) studies. However, due to the small size of our study, we cannot make any inferences on the effect of lead location or stimulation paradigm on the effects of DBS on impulsivity. In addition, while we exact lead locations and trajectories are known to us for 4/8 patients, the remainder had their electrode implantation performed at other institutions and this data is not available.

With these limitations in mind, we have found that the STN adaptively inhibits responses while evidence is being accumulated to make a decision under a state of conflict. Furthermore, it specifically inhibits responses after errors. Here we show that DBS of the STN interrupts both of these mechanisms; DBS prevents filtering of bursts of evidence and it promotes faster responses after errors. The combination of these effects results in impaired decision-making under DBS of the STN.

## Data Availability

The datasets generated for this study can be found in Mendeley Data (https://data.mendeley.com/datasets/3j86m7mjx2/1).

## Ethics Statement

This study was carried out in accordance with the recommendations of the NYU School of Medicine Institutional Review Board with written informed consent from all subjects in accordance with the Declaration of Helsinki. Eligible patients were told about the study at their regularly scheduled appointments and asked if they would be willing to participate.

### Author Contributions

DL, MP, and AM designed the study. MP and AM recruited subjects. DL wrote experimental code, ran experimental sessions, and performed analysis. DL wrote the paper with editing by MP and DL. All authors approved the final manuscript.

### Conflict of Interest Statement

The authors declare that the research was conducted in the absence of any commercial or financial relationships that could be construed as a potential conflict of interest.
